# The Relationship between Health and Community across Aging Cohorts

**DOI:** 10.1155/2014/626097

**Published:** 2014-06-10

**Authors:** Julie Norstrand, Keith T. Chan

**Affiliations:** ^1^Boston College Graduate School of Social Work, McGuinn Hall, Chestnut Hill, MA 02467, USA; ^2^School of Social Welfare, University at Albany, State University of New York, 135 Western Avenue, Richardson 277, Albany, NY 12222, USA

## Abstract

Research is needed to examine the connection between older adults and their community as they age. This is important as increasing numbers of older adults wish to age in place. Regression models were examined across 3 cohorts testing relationships among social capital indicators (neighborhood trust, neighborhood support, neighborhood cohesion, neighborhood participation, and telephone interaction) with health outcomes (self-rated health, activities of daily living (ADL), and instrumental activities of daily living (IADL)). Results showed that most social capital indicators remained significant for all health outcomes into very old age. Development of tools for individual and community interventions to ensure optimal fit between the aging individual and their environment is discussed, along with recommendations for enhancing social work theory and practice.

## 1. Introduction

Interest in age-friendly community and aging-in-place concepts has grown over recent years as the majority of older adults wish to remain within the neighborhood in which they have become familiar and emotionally attached to. Social capital has previously been used to capture community connectedness or social glue [1]. Social capital is relevant to aging-in-place as it provides a framework to examine neighborhoods' fit for older adults; this has important implications for adapting neighborhoods to become more age-friendly and ensure opportunity to aging-in-place. Hence, social capital has gained increased attention from both practice and policy makers [2]. The research has demonstrated a positive relationship between social capital and wide range of health outcomes [3]. In other words, individuals with high social capital are more likely to experience good health. However, most of the research to date has been done on middle-aged adults. It is still not clearly understood how social capital functions in relation to health as adults reach very old age. The goal of this paper was to elucidate this relationship by examining the association between five indicators of social capital with three physical health outcomes among adults aged 65 years and older.

### 1.1. Social Capital

The use of social capital is increasingly recognized as a means for adapting environments for aging-in-place. As Emlet and Moceri argue, an elder friendly community captures the core features of social capital including “civic participation, and the nature of social networks and mutuality/reciprocity” [4, p. 2]. Despite the growing interest in social capital, it remains controversial due to its conceptual polysemy. Therefore, it is important to fully clarify how social capital was conceptualized in this study. Four out of the five social capital indicators used in this study focused on neighborhood (neighborhood trust, neighborhood support, neighborhood cohesion, and neighborhood participation), while the fifth measure (telephone interaction) captured community connections beyond the neighborhood. Therefore, social capital in this study should be considered to be primarily a measure of the neighborhood, with the understanding that social connections can stretch beyond physical borders.

Putnam's definition was used for this study: “features of social organization such as networks, norms, and social trust that facilitate coordination and cooperation for mutual benefit” [5]. There were several reasons for using Putnam's definition; primarily Putnam's conceptualization of social capital has a communitarian focus [6]. A fundamental aspect to communitarianism is the establishment and maintenance of social norms that are fundamental to making communities stronger [7]. Strong communities enable healthier populations; and so Putnam's definition has been widely applied in the health related literature [8]. Since health was the key focus of this study, Putnam's definition of social capital was considered appropriate here. Finally, many of the indicators of social capital available in the dataset used for this study mirror those used to measure social capital as conceptualized by Putnam. Previous research has used a number of these same indicators to reflect social capital [1, 9]. The five indicators used in this study (namely, neighborhood trust, neighborhood support, neighborhood cohesion, neighborhood participation, and telephone interaction) have been used widely in the social capital and health literature [3].

### 1.2. Social Capital and Health among Older Adults

Social capital has been shown to have significant positive associations with a vast array of health outcomes for all ages, including older adults [3, 10]. Kawachi and colleagues [3] found in a number of studies that indicators of social capital are linked to a wide range of health conditions, such as mortality, life-expectancy, self-rated health, cancer, cardiovascular disease, obesity, diabetes, and functional limitations.

Social capital may be particularly important for older adults because this group is “more tethered to their immediate surroundings (and so) the impact of the environment is likely greater” [11, p. 253]. There is growing support for the positive impact of social capital on the health of older adults, even among the oldest-old [12]. In their study, Nyqvist and colleagues found that the* structural* dimension of social capital (i.e., social networks, social integration, and attachment) was important for depressive symptoms among the oldest-old aged 85 and older. The authors argued that this dimension of social capital might be particularly important for this cohort due to their increasing physical frailty and reduced social networks. Therefore, the examination of the relationship between structural dimensions of social capital and health, when conducted across cohorts that represent the changing lifespan of elders (young-old (65–74 years), the middle-old (75–84 years), and the oldest-old (85+ years)), can shed new light on how networks grow, develop, and adapt for this population.

### 1.3. Influence of Age on Social Capital and Health Relationship

Research on whether there are changes in the level of social capital over the lifespan is limited. Research by Axler and colleagues suggested that social capital was the lowest among older adults (aged 60 years and over) compared to younger cohorts [9]. In terms of varying dimensions of social capital, some research has found a decrease as people live into very old age, such as size of social networks and frequency of contacts [13], and civic participation as older adults become less physically active [14]. It is not clear what occurs with other aspects of social capital such as sense of belonging and trust as older adults age. Place attachment, a concept closely related to sense of belonging, does seem to increase with age [15]. However, important physical environmental factors, such as crime, may diminish the sense of belonging [16]. Furthermore, the impact of age on the relationship between social capital and health remains poorly understood. As Cagney and Wen point out: “models of the social capital-health relationship must be attentive to age” [11, p. 239]. Considering the increase in population longevity, the effect of age on the relationship between social capital and health is an important public health query. By understanding the role of age in the relationship between social capital and health, interventions can be developed to optimize the fit between older adults and their community as they age in place.

By partitioning social capital into five indicators, this study was focused on examining how separate dimensions of the social environment can act as determinants of health for older adults, particularly during the latter part of their lifespan. By examining these indicators separately, it is hoped that this will provide a more multifaceted understanding of how elders may feel about their sense of belonging in their neighborhoods and beyond.

### 1.4. Purpose of Study

The relationship among five indicators of social capital (neighborhood trust, neighborhood support, neighborhood cohesion, neighborhood participation, and telephone interaction) which were examined with three health outcomes: self-rated health, activities of daily living (ADL), and instrumental activities of daily living (IADL). Analyses were conducted to examine differences across three cohorts, the young-old (65–74 years), the middle-old (75–84 years), and the oldest-old (85+ years). In order to understand the relationship between social capital and health by age, this study was designed to investigate two questions: (1) does the level of social capital change with age? And (2) does the relationship between social capital and health outcomes change with increasing age? For the first research question we hypothesized that older adults will possess lower levels of certain indicators of social capital (specifically, participation and telephone interaction) while other indicators will increase (specifically, trust, cohesion, and support). As some components of social capital are lost or gained with increasing age, we hypothesized that certain indicators of social capital will have an increased or decreased association with health as individuals progress to a later stage in their lifespan. Specifically, social capital indicators, neighborhood trust, neighborhood cohesion, and neighborhood support, which mirror cognitive dimensions of social capital, will more likely be significantly associated with health among the very old (i.e., oldest-old cohort) compared to younger cohorts. This assumption is based on previous research carried out with the oldest-old, where cognitive dimensions of social capital were found to become increasingly important [12]. The other social capital indicators, neighborhood participation and telephone interaction, will be less likely associated with health outcomes into late adulthood compared to younger cohorts. As described previously, neighborhood participation and telephone interaction have been found to decrease with increased frailty in old age [13, 14].

## 2. Methods

This study used cross-sectional data from the 2010 Community Health Data Base (CHDB) managed by Philadelphia Health Management Corporation. The survey has been conducted biennially since 1994 in five urban and suburban counties of southeastern Pennsylvania (Bucks, Chester, Delaware, Montgomery, and Philadelphia). Random digit dialing (RDD) was used to survey approximately 10,000 households. Persons aged 60 and older were oversampled for the analysis, representing over 700,000 older adults in the area. If a randomly selected adult respondent was unable to be interviewed because of health impairments or language barriers, the interview was conducted with an adult proxy. All respondents who had an adult proxy (*N* = 14, <1%) respond for them were removed from the sample as it was considered important to gain first-hand information from respondents themselves.

## 3. Sample

The sample consisted of 2,344 adults (65 years and older) from a five-county southeastern Pennsylvania region. Just over half (53%) belonged to the young-old cohort, 36% belonged to the middle-old cohort, and 11% belonged to the oldest-old cohort. There were significant differences across the three cohorts across all socioeconomic indicators except by gender. The oldest-old cohort was significantly more likely to be white, have a lower level of education, be poor, and live alone. Marital status varied across cohorts, where older cohorts reported higher percentages of widowed status (Table 1). When comparing this complete sample of older adults to peers nationwide, the percent of older adults (14.5%) was slightly higher than national figures (13%) [17]. However, it should be noted that Pennsylvania (15.4%) had the fourth largest proportion of older adults in 2010 [17]. The complete sample was similar to elders nationwide in terms of age (74.8 years and 74.0 years, resp.). However, generally there were fewer females, minority, and lower level of educational attainment among elders nationwide [18] compared to the sample examined in this study. Poverty (@ 100% FPL) among elders nationwide (9.0%) was higher than the complete sample (7.6%) [18]. However, there was considerable variation in poverty level in the complete sample by county. For example, 13.6% older Philadelphians were poor [19].

## 4. Measures

### 4.1. Physical Health Outcomes

*Self-Rated Health.* This was measured by a single item that asked to rate health on a 5-point Likert scale, with a high number indicating better self-rated health.

*ADL and IADL.* ADL reflected eight basic activities that generally occur within the home (i.e., eating, dressing, grooming, walking, transferring, bathing, continence, and soiling). IADL measured more complex tasks needed for the ADLs that generally require interaction with the surrounding environment (i.e., talking on the phone, walking, shopping, meal preparation, housework, taking medicine, and handling money). For this study, ADL and IADL scales were dichotomized with 0 representing no ADL/IADL limitations and 1 representing 1 or more ADL/IADL limitations. In this study, only 10.7% had 1 or more ADL and 25.4% had 1 or more IADL, similar to national statistics from other large-scale studies from the CDC and the US Census. Conceptually, having 1 or more limitations with ADLs or IADLs can have a direct impact on an older person's ability to age in place.

### 4.2. Social Capital Indicators

The five social capital indicators were originally derived from the Social Capital Community Benchmark Survey [20]. The items were as follows.

*Neighborhood Support*. This was assessed by “please rate how likely people in your neighborhood are willing to help their neighbors with routine activities such as picking up their trash cans, or helping to shovel snow. Would you say that most people in your neighborhood are always, often, sometimes, rarely, or never willing to help their neighbors?” Response categories were recoded from 1 to 4, with 1 being rarely/never, 2 being sometimes, 3 being often, and 4 being always.

*Neighborhood Participation*. This was assessed by “How many local groups or organizations in your neighborhood do you currently participate in such as social, political, religious, school-related, or athletic organizations?” Responses categories ranged from 0 to 12 groups. Due to very small number of cases in category responses 6 and higher, this variable was top-coded so response categories ranged from 0 to 6.

*Neighborhood Cohesion*. This was assessed by “Please tell me if you strongly agree, agree, disagree, or strongly disagree with the following statement: I feel that I belong and am a part of my neighborhood.” Responses categories were coded from 1 to 4 with 1 being strongly disagree, 2 being disagree, 3 being agree, and 4 being strongly agree.

*Neighborhood Trust*. This was assessed by “Please tell me if you strongly agree, agree, disagree, or strongly disagree with the following statement: Most people in my neighborhood can be trusted.” Response categories were also coded from 1 to 4 with 1 being strongly disagree, 2 being disagree, 3 being agree, and 4 being strongly agree.

*Telephone Interaction*. This was assessed by “About how often do you talk with friends or relatives on the telephone?” Response categories included several times a day, once a day, a few times a week, once a week, less often than once a week, and never. Responses categories were recoded from 1 to 4, with 1 being once a week or less, 2 being few times a week, 3 being once a day, and 4 being several times a day.

### 4.3. Demographic and Socioeconomic Covariates

Demographic and socioeconomic variables entered into the analyses were* age* in years ranging from 65 and higher;* sex*, 0 representing female and 1 representing male;* race*, a dichotomized variable with 0 representing white and 1 representing minority, (minority includes all nonwhite plus all Hispanics of any race);* education*, coded along 5 response categories: less than high school graduate (0–11 years), high school graduate (12 years), some college (13–15 years), and postcollege (more than 16 years) with high school (16 years) as comparison group;* poverty* at 200% of the federal poverty line dichotomized into poor (coded 1) and nonpoor (coded 0);* marital status*, recoded into 4 dummy variables (single, divorced, and widowed with married as comparison group); and* living arrangements*, a dichotomized variable representing living alone (coded 0) versus living with others (coded 1). Poverty at 200% was used because it represented a more reasonable cut-off for poverty than 100% [21].

## 5. Data Analysis

The first research question was tested using Kruskal-Wallis rank sum tests on the five social capital indicators (neighborhood trust, neighborhood support, neighborhood cohesion, neighborhood participation, and telephone interaction) with the sample (>65 yrs) split along by three cohorts (65–74, 75–84, and 85 and older). Cohort analysis was used to examine differences across the lifespan of older adults, where important differences may emerge for the oldest-old (85 and older), who are the most at risk. The second research question was tested using binary logistic (ADL and IADL) and ordinal logistic regression (self-rated health), analyses for each of the health outcomes as separate dependent variables for each of the three cohorts. Standard socioeconomic indicators were accounted for as covariates in the analyses.

## 6. Results

### 6.1. Research Question 1

Findings suggest that age did not play a role in terms of possession of social capital except for neighborhood support (Table 1). Results from Kruskal-Wallis rank sum tests showed significant differences across cohorts for the indicator neighborhood support. The rank sum was the highest for the youngest group and became gradually lower as respondents aged (65–74: 1,240,000; 75–84: 887,372; 85+: 251,588), suggesting that support from neighbors as an indicator of social capital deteriorates as participants progressed to a later lifespan.

Differences across the three cohorts were more notable for health (Table 2). Significant differences were found on all four health outcomes across the three groups, with worsening health profiles as age increased. Post hoc tests showed significant differences between all groups for ADL and IADL. Significant differences were found between the young-old and oldest-old for self-rated health. Significant differences in terms of sociodemographic characteristics were also found across groups (Table 3). As age increased, the sociodemographic profile was more likely to be white, less educated, poor, and living alone.

### 6.2. Research Question 2

Tables 4, 5, and 6 showed the results of the logistic regressions carried out for each of the health outcomes across the three cohorts. Generally the fit of these regression models was significant for health outcomes across all three groups, except for self-rated health and ADL among the oldest-old. Despite the limited variance explained by the models, the results showed a complex set of associations between social capital indicators and health outcomes that changed with age. All of the social capital indicators, except telephone interaction, were significantly associated with all of the health outcomes. Interestingly, these social capital indicators were relevant at different stages of the lifespan. Specifically, for the health outcome self-rated health (Table 4) neighborhood cohesion improved health among the young-old (OR = 1.31, *P* < 0.05), while neighborhood trust (OR = 1.87, *P* < 0.001) and neighborhood participation (OR = 1.14, *P* < 0.05) improved health among the middle-old. For the middle-old, respondents who reported greater neighborhood trust (OR = 0.63, *P* < 0.05) and neighborhood participation (OR = 0.74, *P* < 0.05) were around 30% less likely to have an ADL limitation (Table 5). No significant associations were observed for social capital indicators among the young-old for ADL. In terms of IADL limitations (Table 6), the oldest-old group with more neighborhood cohesion were 50% less likely to have an IADL limitation (OR = 0.45, *P* < 0.05). On the other hand, those who were 85 and older who reported higher levels of neighborhood trust were twice as likely to report an IADL limitation (OR = 2.00, *P* < 0.05). Social capital indicators were not observed to be significant with IADL limitations for the two other cohorts.

## 7. Discussion

We hypothesized that certain indicators of social capital would increase (namely, neighborhood trust, neighborhood cohesion, and neighborhood support) or decrease (namely, neighborhood participation and telephone interaction) with increasing age. The trends in the percentage breakdowns did show some support for this (Table 2). For example, the oldest-old reported lower levels of both neighborhood participation and telephone interaction than their younger cohorts. Also, the oldest-old also reported higher levels of trust than younger cohorts. Neighborhood cohesion did not follow the expected trend as it decreased with age. These findings add to the limited literature on the change in social capital over the lifespan. In terms of support, our findings found the perception that neighbors are willing to help did increase to a certain age. According to our study, middle-old cohort reported the highest level of neighborhood support. What is interesting to note about this cohort is that they possessed high (or higher) levels of neighborhood support, neighborhood cohesion, and neighborhood trust compared to the other two cohorts. This could suggest that the neighborhood, in terms of support, cohesion, and trust, may be particularly meaningful for this cohort. Other indicators of social capital examined in this study (i.e., neighborhood participation and telephone interaction) did not follow this trend. For example, the middle-old cohort reported the lowest frequency of telephone interactions with friends, relatives, and neighbors, although this difference was not significant. The variation in possession of different indicators of social capital by cohort could be used in terms of developing communities that maximize the potential for successful aging-in-place; such policy implications are described further below.

Logistic regressions for each of the four health outcomes showed that different indicators of social capital continued to be significant predictors of health outcomes even when accounting for standard sociodemographic predictors; however, the nature of these associations changed with increasing age. With respect to the second hypothesis, our expectation was supported in the fact that different indicators of social capital were relevant to health as age increased. We had specifically predicted that the social capital indicators, neighborhood trust, neighborhood cohesion, and neighborhood support, would more likely be associated with improved health among those of advanced age (i.e., the oldest-old cohort) compared with the younger cohorts (i.e., young-old). This prediction did bear out for IADL with respect to neighborhood trust and neighborhood cohesion for the oldest-old. However, the relationship was in the opposite direction with neighborhood trust. It is likely that this finding reflects endogeneity in the relationship of neighborhood trust and reporting an IADL limitation; elders who are among the oldest and most vulnerable must trust the people around them to even be able to seek out help for complex tasks with negotiating the outside world. We also predicted that the social capital indicators, neighborhood participation and telephone interaction, would be less likely to be significant with increasing age. The results indicated that there was no relationship to health for these variables.

Albeit the fact that social capital explained a very small part of the overall variance of each of the health outcomes, what is important to derive from these findings is how different aspects of the social environment matter for different health outcome at different stages of life. Specifically, neighborhood trust was only significant among the middle-old cohort for self-rated health and ADL, whereas neighborhood trust had an unexpected relationship with IADL limitations for the oldest-old. Neighborhood cohesion was significant for the young-old for self-rated health whereas neighborhood cohesion was significant for the oldest-old in terms of IADL. Neighborhood support was significant for the oldest-old in terms of self-rated health. Finally, neighborhood participation was only significant for the middle-old for self-rated health and ADL. This mixed pattern of associations suggests that all parts of the social environment play a role across the aging lifespan and for different indicators of health. This is a significant finding suggesting that the social environment is a critical factor to be considered when adapting settings for aging-in-place. This also highlights the importance of looking at social capital in terms of multiple indicators rather than creating a single factor. Furthermore, these findings also suggest that the “age structure of the community” also needs to be taken into account [11, p. 251]; in other words, the social environment must fit the needs of the age demographics.

The nature of the relationship between the five social capital indicators and the three health outcomes may provide some interesting insight into the meaning of these health outcomes in relation to the social environment. Compared to ADL and IADL, the relationship between social capital and self-rated health was noted across all three age groups. Both self-rated health and social capital are highly subjective concepts; and it is possible that subjective processes applied to each of these domains (i.e., self-rated health and social capital) influence one another. In terms of functional health, social capital was significant only among the oldest-old for IADL whereas it was significant only among the middle-old for ADL. It is important to pay attention to which dimensions of the social environment mattered: among the oldest-old, neighborhood support and neighborhood trust were critical for IADL. Among the middle-old, neighborhood trust and neighborhood participation were significant. Although these patterns of relationships do make sense, it is difficult to explain why social capital did not matter for either ADL or IADL for the youngest-old. Perhaps grandparent/family responsibilities diminish as people move from young-old to middle-old age. This may give them more time to engage with neighbors; and this builds familiarity (experienced as trust and sense of belonging) with the neighborhood. As Kahn and Antonucci [22] Convoy model explicates, relationships change over the lifespan that can impact health and well-being. More research is needed to better understand what and how older adults interact with their neighborhoods.

The associations between social capital indicators and health outcomes were in the expected direction; in other words, high levels of social capital indicators were associated with better health outcomes. There was one exception though, and that was with the relationship between neighborhood trust and IADL limitation among the oldest-old. Among this cohort, higher neighborhood trust was associated with higher IADL limitations. This was an unexpected finding. It may be that individuals who experienced high levels of IADL limitations required help from neighbors and thus develop higher levels of trust through this interaction. This interpretation does bring up an important point regarding the need for caution when making assumptions about the direction of the relationship between social capital and health.

The one indicator of social capital that was not significant for any of the four health outcomes examined in this study was telephone interaction. The principal author also found similar findings in a previous study; in that study it was noted that it may be not the frequency of contact (as was measured in this study) but rather the level of support provided by the network that was the critical factor for physical and mental health [10]. Lack of significant association for telephone interaction with any of the three health outcomes in this study may provide some insight into social capital: it is quality of connection rather than quantity of connections within the neighborhood that is important for social capital to capture. This finding may also pertain to other dimensions of social capital, such as neighborhood participation. In other words, the quality of neighborhood participation (i.e., measuring the type of activity) rather than the number of organizations that the individual is participating with might better capture the benefits of social capital. Ultimately, the findings in this study suggest that more attention needs to be paid to the perception of community and the people living in these communities. Indeed, the role of neighbors is likely to increase in the future as the availability of spouses or children to provide care for aging parents is expected to diminish. This is due to reduction in fertility rates, increased divorces rates, and low rates of remarriage [23, 24].

## 8. Conclusions

It is hoped that the findings of this research may build on optimal environments for aging-in-place and elder-friendly communities by furthering our understanding of what dimensions of the social environment matter for health. As Emlet and Moceri have argued, there is a need “to further elucidate the importance of social relationships and social connectedness with aging in place and in developing elder friendly communities” ([4, p. 3]). It was the aim of this research to provide a comprehensive empirical examination of the fit between the aging individual and their surrounding environment in relation to health.

The dynamic between the aging individual and the community in which they live is a complex one. Although previous research has described an important role of the environment in which older adults live, this study has provided rich empirical evidence for this by examining relationships between* multiple* indicators of social capital and health across three cohorts. It is hoped that these findings will encourage the development of programs and interventions that allow for greater exposure to these important indicators of the social environment among older adults. Indeed, the results suggest that there is opportunity for intervention at numerous points throughout the end of the lifespan, and different dimensions of the social environment may be more critical at different points of the lifespan. For example, this study showed that neighborhood support was important for self-rated health among the oldest-old. A possible means of building support could be through programs that help establish connections between neighbors who have volunteered to assist with those who need assistance. Although this type of intervention may appear intrusive, the need for assistance among elders is increasingly recognized and accepted by elders themselves, as well as by family who live at a distance. Additionally, neighbors may not mind helping others if they understand that there is a need, and this is part of the problem they can help to address in their proverbial backyards. Such awareness has grown after infamous events such as the heat waves in Chicago in 1995 [25] and in Paris in 2003 [26] where rates of death were particularly high among the frail elderly. These events highlighted the need for greater awareness of and reaching out to frail neighbors. Neighbors providing assistance present as an alternative to cost-prohibitive home care. Interventions directed at the oldest-old could also incorporate neighborhood trust and neighborhood cohesion, as these were indicators of social capital found to be critical for IADL of the oldest-old. Interventions focused on the oldest-old are mentioned here, yet it is important to point out that each of the indicators of social capital (except telephone interaction) could be incorporated into interventions directed at the young-old and middle-old as well.

From a lifespan perspective, an important question to ask here is when should interventions aimed at increasing social capital be made? For example, should an effort be made to intervene during the middle-old aged years or earlier? Ideally interventions aimed at augmenting an individual's social capital should be implemented early in life, during youth as research has shown that children that volunteer are more likely to volunteer in adulthood and later in life (Oesterle, Johnson, and Mortimer, 2004). Perhaps interventions aimed at building social capital specifically among older adults should be done at or before the point when retirement is being considered. It is possible to conceive of a program, which at the exit job interview puts the individual, who is soon to retire, in connection with volunteer organizations in the community. In this way, the program helps build a bridge between the individual and their community, and so the older adult can start to build social capital through volunteering in the community.

When considering interventions aimed at building social capital, the geographic context should also be taken into account. The sample of older adults in this study resided is urban and suburban settings of five counties of southeastern Pennsylvania. Philadelphia, the county with the highest proportion of older adults (namely, 39% of sample), represented a highly urbanized context while the other four counties reflected mostly suburban settings. Pathways that link social capital and health may differ according to geographic context [27]. These authors describe characteristics of urbanization such as crime, minority, inequality, and population stability that may influence social capital and health. Specifically, Pridmore and colleagues write, “Poor urban settlements require special interventions to address social exclusion, disruption of social networks and trust, insecurity and violence, and high mortality and morbidity” [27, p. i138]. Ultimately, the context in which the individual lives requires consideration when developing interventions aimed at building social capital. Furthermore, the level at which the intervention is to be provided must also be taken into account. For example, the approach will be different if the intervention is to occur at the individual or microlevel, city or mesolevel, or the macrolevel.

### 8.1. Limitations of the Study

An important limitation of this study is that the data is based on a cross-sectional design. This means that definitive statements about causality cannot be made. Most likely the association between social capital and health is bidirectional, as has been demonstrated in previous research [28]. Some additional limitations to be considered when interpreting the findings: first and foremost, size of the sample for the oldest-old was fairly small (*N* = 266) compared to the young-old (*N* = 1233) and the middle-old (*N* = 845). These sample sizes were further reduced in the regression models. Small sample size of the oldest-old cohort may have limited the power of the statistical significance tests. This may explain the nonsignificant models for self-rated health and ADL for oldest-old. This also brings up the important consideration regarding survival. In other words, those examined in this study represented those who were by default healthier and had chosen to stay aging in place. Another limitation was that the definition and measurement of social capital remain elusive. As was found in this study, not all the measures used were associated with health, specifically telephone interaction. We continue to need a better understanding of how to best measure social capital. Furthermore, as Nyqvist and colleagues have stated:, “It is generally assumed that social capital indicators measure the same thing in different groups and places” [12, p. 105]. However, research has demonstrated that there are marked differences in the questions about social capital that are considered appropriate for various groups depending, for instance, on the subjects' age [29].

It is also important to note that the effect sizes for the analyses were somewhat low, given the small *R* ^2^ findings. However, because this study employed nonparametric techniques for estimation, it can be problematic to rely too heavily on these measures of linear fit. Despite these concerns, the results do clearly indicate that community-level neighborhood social capital does have a relationship to health for elders, even when including important individual-level factors. This speaks to the mezzo- and macrolevel implications of these findings and warrants further investigation in research.

### 8.2. Considerations for Future Research

This research clearly calls for use of longitudinal data as this could provide answers regarding causality as age increases, in the hope of developing age-sensitive interventions across the lifespan. These findings also need to be replicated with larger sample sizes, especially for the oldest-old group. Furthermore, as highlighted above, the geographic context needs to be given greater focus in future research. Also, in response to Nyqvist and colleagues' concerns, there is a need for social capital scale development that reflects the specific characteristics of older persons. Furthermore, questions asking whether residents plan to move in the near future and why should be included in order to get a better understanding of how the social environment might impact decisions leading to moving away from one's neighborhood. Finally, based on the findings of this study certain dimensions of the social environment (namely, trust, cohesion, support, and participation) were found to be associated with health at different parts of the aging lifespan. However, the question still remains as how does one build, for example, trust and cohesion? Successful implementation of programs that build social capital should also take into consideration individual characteristics, such as gender, race, and economic status that may influence the relationship between social capital and health. Finally, impact of social capital on the individual needs to be examined in the light of Lawton's environmental docility and environmental proactivity distinction. In other words, as age progresses how do different dimensions of the social environment either benefit or impede functioning? Ultimately, there continues to be a need to refine the person-environment fit model over the lifespan [30].

## Figures and Tables

**Table 1:** Socioeconomic characteristics by cohort.

	65–74 yrs (*N* = 1233) (%)	75–84 yrs (*N* = 845) (%)	85+ yrs (*N* = 266) (%)	*χ* ^2^ (df)	*P*
Gender (female)	67	69	69	1.12 (2)	0.60
Race (nonwhite)	26	20	17	17.33 (2)	0.001
Education (<HS)	10	13	15	26.66 (8)	0.001
Poverty (200% FPL)	28	34	41	22.46 (2)	0.001
Marital status				268.21 (6)	0.001
Married/living with someone Widowed Divorced/separated Single	51 20 16 13	36 47 8 10	25 61 4 10		
Living arrangements (alone)	37	48	66	86.16 (2)	0.001

**Table 2:** Social capital characteristics by cohort.

	65–74 yrs (*N* = 1233) (%)	75–84 yrs (*N* = 845) (%)	85+ yrs (*N* = 266) (%)	*χ* ^2^ (df)^a^	*P*
SC: cohesion				16.97 (6)	0.009
Strongly disagree Disagree Agree Strongly agree	1 6 60 34	0 4 64 31	2 9 58 31		
SC: support				15.84 (6)	0.015
Never/rarely Sometimes Often Always	11 26 32 31	11 22 28 38	15 22 27 36		
SC: Trust				5.32	0.503
Strongly disagree Disagree Agree Strongly agree	2 10 62 26	2 9 66 23	1 8 62 29		
SC: participation				25.67	0.177
0 1 2 3+	44 26 15 15	45 26 13 16	49 27 14 10		
SC: telephone interaction				5.87	0.438
Once a week Few times a week Once a day Several times a day	10 25 23 42	12 28 22 38	10 27 25 38		

^a^The Kruskal-Wallis statistic, a test of rank for nonparametric samples, was also conducted for all social capital items to examine differences in the 3 groups. Results indicated statistical differences for support (KW = 6.59, df = 2, and *P* = 0.04), but no differences for cohesion, trust, and participation (cohesion: KW = 1.58, df = 2, and *P* = 0.46; trust: KW = 1.66, df = 2, and *P* = 0.44; telephone interaction: KW = 3.71, df = 2, and *P* = 0.16). In the case of support, the cohort of 65 to 74 was ranked the highest for support (rank sum = 1,240,000), followed by those who are 75 to 84 (rank sum = 887,372), and last for those who are 85 and older (rank sum = 251,588).

**Table 3:** Health outcomes by cohort.

	65–74 yrs (*N* = 1233) (%)	75–84 yrs (*N* = 845) (%)	85+ yrs (*N* = 266) (%)	*χ* ^2^ (df)	*P*
Self-rated health^a^				33.03	0.000
Poor Fair Good Very good Excellent	4.07 16.12 33.96 30.37 15.47	7.13 17.34 36.82 27.43 11.28	7.52 24.81 32.33 24.81 10.53		
No ADL	92.94	88.17	77.82	55.81 (2)	0.000
No IADL	83.94	69.47	51.13	147.22 (2)	0.000

^a^The Kruskal-Wallis statistic, a test of rank for nonparametric samples, was also conducted for each self-rated health to examine differences in the 3 groups. Results for the KW indicated significant differences in self-rated health between groups (KW = 23.194, df = 2, and *P* < 0.0001), with those who are 65 to 74 having the highest health (rank sum = 1,510,000), followed by those who are 75 to 84 (rank sum = 943,626), and those who are 85 and older having the lowest health (rank sum = 277,376).

**Table 4:** Self-rated health with all predictors (odds ratios with 95% interval confidence).

Age category	65–74 yrs		75–84 yrs		85+ yrs	
Predictor	OR	95% C.I.	OR	95% C.I.	OR	95% C.I.
Age	1.00	0.96; 1.04	0.92***	0.87; 0.97	1.06	0.96; 1.17
Sex (male)	0.81	0.62; 1.04	0.76	0.56; 1.05	0.72	0.37; 1.38
Race (minority)	0.57***	0.43; 0.76	0.59**	0.41; 0.87	0.69	0.31; 1.55
Education	1.34***	1.20; 1.49	1.17*	1.02; 1.35	1.19	0.89; 1.58
Poverty @ 200% (poor)	0.49***	0.37; 0.66	0.61**	0.44; 0.87	1.40	0.75; 2.62
Marital status (married)						
Widowed	0.59*	0.40; 0.87	0.91	0.59; 1.41	1.25	0.55; 2.86
Divorced/separated	0.48**	0.32; 0.74	1.18	0.59; 2.38	0.56	0.12; 2.55
Single	0.50*	0.32; 0.77	1.07	0.58; 1.94	0.98	0.34; 2.83
Living arrangement (with others)	0.76	0.54; 1.06	0.66*	0.44; 0.99	1.59	0.78; 3.24
SC: neighborhood cohesion	1.31*	1.04; 1.65	1.10	0.82; 1.48	1.15	0.71; 1.89
SC: neighborhood support	1.05	0.92; 1.19	1.11	0.95; 1.30	1.35*	1.01; 1.80
SC: neighborhood trust	1.06	0.87; 1.31	1.87***	1.42; 2.46	1.23	0.76; 1.97
SC: neighborhood participation	1.05	0.96; 1.14	1.14*	1.02; 1.26	1.09	0.94; 1.26
SC: telephone interaction	1.02	0.91; 1.14	0.90	0.79; 1.04	0.93	0.71; 1.23
Model fit						
*R* ^2^	0.07		0.05		0.04	
*F*(df)	*F*(14,997) = 194.82**		*F*(14,641) = 94.13*		*F*(14,186) = 20.94	

***P ≤ 0.001; **P ≤ 0.01; *P ≤ 0.05.

**Table 5:** ADL with all predictors (odds ratios with 95% interval confidence).

Age category	65–74 yrs		75–84 yrs		85+ yrs	
Predictor	OR	95% C.I.	OR	95% C.I.	OR	95% C.I.
Age	0.97	0.89; 1.07	1.20***	1.09; 1.31	1.08	0.95; 1.23
Sex (male)	0.66	0.35; 1.24	0.81	0.46; 1.43	0.78	0.31; 1.95
Race (minority)	1.90*	1.08; 3.31	2.13**	1.20; 3.78	0.30	0.07; 1.18
Education	1.06	0.82; 1.37	1.07	0.84; 1.37	1.16	0.78; 1.73
Poverty @ 200% (poor)	3.64***	1.98; 6.71	2.08*	1.17; 3.69	1.89	0.76; 4.68
Marital status (married)						
Widowed	0.94	0.41; 2.17	1.61	0.79; 3.28	0.73	0.21; 2.48
Divorced/separated	1.20	0.50; 2.86	3.12*	1.08; 9.05	1.67	0.27; 10.44
Single	1.45	0.61; 3.46	1.70	0.59; 4.85	1.26	0.31; 5.21
Living arrangement (with others)	0.87	0.45; 1.69	2.07*	1.08; 3.95	1.13	0.38; 3.43
SC: neighborhood cohesion	0.97	0.57; 1.64	1.33	0.80; 2.21	0.74	0.38; 1.46
SC: neighborhood support	1.08	0.81; 1.43	0.90	0.69; 1.17	0.95	0.63; 1.43
SC: neighborhood trust	0.85	0.55; 1.30	0.63*	0.41; 0.98	0.89	0.47; 1.70
SC: neighborhood participation	1.03	0.84; 1.26	0.74*	0.58; 0.94	1.05	0.85; 1.30
SC: telephone interaction	0.94	0.73; 1.20	1.06	0.83; 1.36	0.96	0.66; 1.39
Model fit						
*R* ^2^	0.09		0.11		0.06	
*F*(df)	*F*(14,999) = 44.65***		*F*(14,644) = 50.05***		*F*(14,186) = 11.83	

***P ≤ 0.001; **P ≤ 0.01; *P ≤ 0.05.

**Table 6:** IADL with all predictors (odds ratios with 95% interval confidence).

Age category	65–74 yrs		75–84 yrs		85+ yrs	
Predictor	OR	95% C.I.	OR	95% C.I.	OR	95% C.I.
Age	1.00	0.94; 1.06	1.15***	1.08; 1.22	1.12	0.99; 1.27
Sex (male)	0.75	0.48; 1.15	0.80	0.54; 1.20	0.45*	0.20; 0.99
Race (minority)	1.74**	1.17; 2.59	1.71*	1.09; 2.69	0.92	0.37; 2.32
Education	0.88	0.74; 1.06	1.03	0.87; 1.23	1.01	0.71; 1.44
Poverty @ 200% (poor)	2.15***	1.42; 3.26	1.74**	1.15; 2.64	1.48	0.68; 3.22
Marital status (married)						
Widowed	1.79*	1.02; 3.14	1.79*	1.06; 3.02	1.83	0.65; 5.16
Divorced/separated	1.51	0.81; 2.82	1.98	0.86; 4.55	2.80	0.53; 14.96
Single	1.99*	1.07; 2.73	1.50	0.70; 3.24	4.41*	1.13; 17.24
Living arrangement (with others)	1.25	0.78; 2.00	1.79*	1.10; 2.92	1.22	0.50; 2.98
SC: neighborhood cohesion	0.79	0.55; 1.14	1.17	0.81; 1.68	0.45*	0.23; 0.85
SC: neighborhood support	1.13	0.92; 1.37	0.83	0.69; 1.00	0.70	0.49; 1.01
SC: neighborhood trust	0.87	0.64; 1.18	0.94	0.68; 1.31	2.00*	1.08; 3.70
SC: neighborhood participation	0.97	0.83; 1.11	0.89	0.77; 1.02	0.94	0.76; 1.17
SC: telephone interaction	0.99	0.83; 1.19	1.08	0.91; 1.29	1.06	0.76; 1.46
Model fit						
*R* ^2^	0.09		0.06		0.14	
*F*(df)	*F*(14,999) = 77.37***		*F*(14,644) = 50.38***		*F*(14,186) = 35.75***	

***P ≤ 0.001; **P ≤ 0.01; *P ≤ 0.05.
